# In Vitro Assessment of Wound-Healing Efficacy of Stabilized Basic Fibroblast Growth Factor (FGF-2) Solutions

**DOI:** 10.3390/ph17020247

**Published:** 2024-02-14

**Authors:** Leah Benington, Jingxin Mo, Mingxin Li, Gunesh Rajan, Cornelia Locher, Lee Yong Lim

**Affiliations:** 1 Division of Pharmacy, School of Allied Health, University of Western Australia, Perth, WA 6009, Australia; leah.benington@uwa.edu.au (L.B.); connie.locher@uwa.edu.au (C.L.); 2 Neuroscience Laboratory, The Affiliated Hospital of Guilin Medical University, Guilin 541001, China; jingxin.mo@hotmail.com (J.M.); mingxinli822@163.com (M.L.); 3 Graduate School of Biomedical Engineering, University of New South Wales, Sydney, NSW 2052, Australia; 4 Department of Pharmacy, Tangshan Central Hospital, Tangshan 063000, China; 5 Otolaryngology, Head & Neck Surgery, Division of Surgery, Medical School, University of Western Australia, Perth, WA 6009, Australia; gunesh.rajan@uwa.edu.au; 6 Department of Otolaryngology, Head & Neck Surgery, Luzerner Kantonsspital, 6000 Luzern, Switzerland

**Keywords:** fibroblast growth factor 2, basic fibroblast growth factor, stabilisation, thermal stability, processing stability

## Abstract

Chronic tympanic membrane perforations (TMP) pose a significant clinical challenge, but basic fibroblast growth factor (FGF-2) shows promise for their treatment, despite its instability in aqueous solutions which hampers the sustained delivery crucial for the healing process. Addressing this, our research focused on the development of stabilized FGF-2 formulations, F5 and F6, incorporating dual, generally regarded as safe (GRAS) excipients to enhance stability and therapeutic efficacy. F5 combined FGF-2 (1600 ng/mL) with 0.05% *w*/*v* methylcellulose (MC) and 20 mM alanine, while F6 used FGF-2 with 0.05% *w*/*v* MC and 1 mg/mL human serum albumin (HSA). Our findings demonstrate that these novel formulations not only significantly improve the cytoproliferation of human dermal fibroblasts but also exhibit the most potent chemoattractant effects, leading to the highest fibroblast monolayer closure rates (92.5% for F5 and 94.1% for F6 within 24 h) compared to other FGF-2 solutions tested. The comparable performance of F5 and F6 underscores their potential as innovative, less invasive, and cost-effective options for developing otic medicinal products aimed at the effective treatment of chronic TMP.

## 1. Introduction

Tympanic membrane perforation (TMP) is commonly caused by ear infection, mechanical trauma, or a pressure blast injury [1,2]. Most TMP will repair without medical treatment. However, some do not heal, with chronic perforations causing serious problems such as recurrent ear infections and hearing loss [3]. Chronic TMP are managed surgically with myringoplasty and tympanoplasty, both of which are expensive and intrusive grafting procedures with success rates that vary widely from 35% to 94% [1,4]. A significant number of patients require repeat surgery, and even when the surgery is adequate, the grafted membrane is often acoustically suboptimal and prone to re-perforation [5,6,7].

The application of exogenous growth factors known to stimulate cellular regenerative processes [1,8,9,10] may provide a cost-effective, less invasive, and reliable treatment for chronic TMP. The basic fibroblast growth factor (FGF-2) is one such promising agent. An endogenous chemoattractant [11], FGF-2, promotes the cytoproliferation of endothelial, epithelial, preadipocyte, fibroblast and stem cells [12,13,14]. Clinically, it has yielded positive outcomes as a wound-healing agent for tissues with multiple cell types, including the tympanic membrane [15,16,17,18].

The clinical translation of FGF-2 has, however, proven difficult, mainly because of its extremely poor stability in aqueous media. In one study, FGF-2 was shown to degrade so rapidly it was difficult to detect and quantify bioactive FGF-2 in cell culture samples [16,17,18,19,20,21]. Most successful clinical studies investigating FGF-2 for chronic TMP required daily applications of freshly prepared FGF-2 formulations as its bioactivity lasts for only 24–36 h [22,23,24]. None of the studies published information on the bioactive FGF content or the stability of the in situ compounded-FGF-2 formulations administered to trial participants. Moreover, the FGF-2 doses applied in these studies varied between 5 and 30 µg [22,23,25,26], a 6-fold difference, and it might be speculated that the higher applied doses were required due to the instability of FGF-2 in solution. Administration of high FGF-2 doses by patients, as well as repeated applications, are not desirable as they can lead to adverse effects that include secondary otitis media and membrane re-perforation [27]. On this basis, it will be helpful to design a stable otic FGF-2 medicinal product that allows the healing of chronic TMP to be achieved safely and predictably using a patient-centred dosing regimen.

We have previously reported that FGF-2 in solution is stabilized by incorporating into the solution methylcellulose, alone or in combination with alanine or human serum albumin, all of which are GRAS (generally regarded as safe) pharmaceutical excipients [28]. The excipients also fully protect FGF-2 against degradation when it is exposed to thermal and processing stressors encountered in its transformation from solution into a powder formulation by freeze drying. FGF-2 content in the stabilized formulations were confirmed via ELISA; however, it is not yet known if the stabilized FGF-2 formulations would provide efficacious wound healing. To achieve the full potential of the stabilized formulations, it is essential to prove that the added excipients do not negatively impact the cytoproliferative activity of FGF-2 that underpins its capacity to heal chronic TMP. The aims of this present study are to demonstrate via an in vitro model of dermal human fibroblasts that the stabilized FGF-2 formulations can produce cytoproliferative and chemotactic migration effects, and that these effects are more sustained and superior to FGF-2 solution without the stabilizers.

## 2. Results

### 2.1. Dose-Response Assay

**Figure 1:**
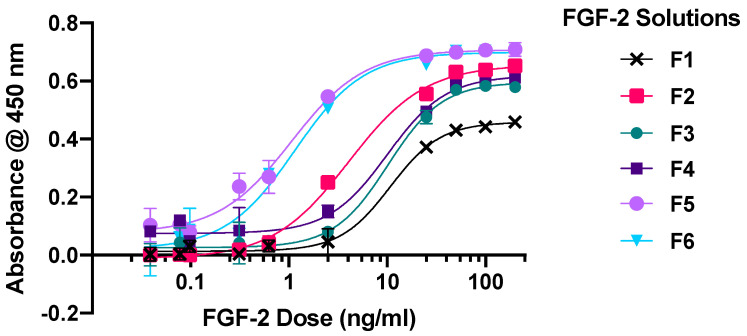
Cellular proliferation curves of primary human dermal fibroblasts in response to escalating doses (0.0098–200 ng/mL) of FGF-2 samples: F1 (control), F2 (with methylcellulose (MC) 0.05% *w*/*v*), F3 (alanine 20 mM), F4 (human serum albumin (HSA) 1 mg/mL), F5 (MC 0.05% *w*/*v* and alanine 20 mM) and F6 (MC 0.05% *w*/*v* and HSA 1 mg/mL). Cytoproliferative effects were measured via a CCK-8 assay. Data represent mean ± SD (*n* = 3).

Six solutions containing 1600 ng/mL FGF-2 as measured by ELISA assay were prepared with different excipients and diluted appropriately to give concentrations of 0.0098 to 200 ng/mL for the cytoproliferative experiments. F1 was without excipient (control), F2, F3 and F4 contained only MC, alanine, or HSA, respectively, while F5 contained MC in combination with alanine, and F6 contained MC in combination with HSA. All six solutions showed sigmoidal dose-response curves with poor cytoproliferative effects at the lowest 0.0098 ng/mL FGF-2 and a plateauing of effects at FGF-2 doses ≥ 50 ng/mL (Figure 1). The solutions with the stabilising excipients (F2–F6) produced greater cytoproliferation of the dermal fibroblasts than the control F1 at FGF-2 concentrations between 25 and 200 ng/mL (*p* < 0.0001); the maximal activity ranking was in the decreasing order of F5~F6 > F2 > F4~F3 > F1. Moreover, the EC_50_ of F5 and F6 (mean 1.064 ng/mL and 1.145 ng/mL, respectively) was lower compared to that of F2 (4.104 ng/mL), and even more so when compared to F1 (10.754 ng/mL), F3 (10.191 ng/mL) and F4 (10.17 ng/mL). F5 and F6 showed comparable effectiveness over the concentration range of 50–200 ng/mL (*p* = 0.8399); however, F5 was more effective than F6 at lower FGF-2 concentrations (0.098–2.5 ng/mL).

### 2.2. Wound-Healing Assay

**Figure 2:**
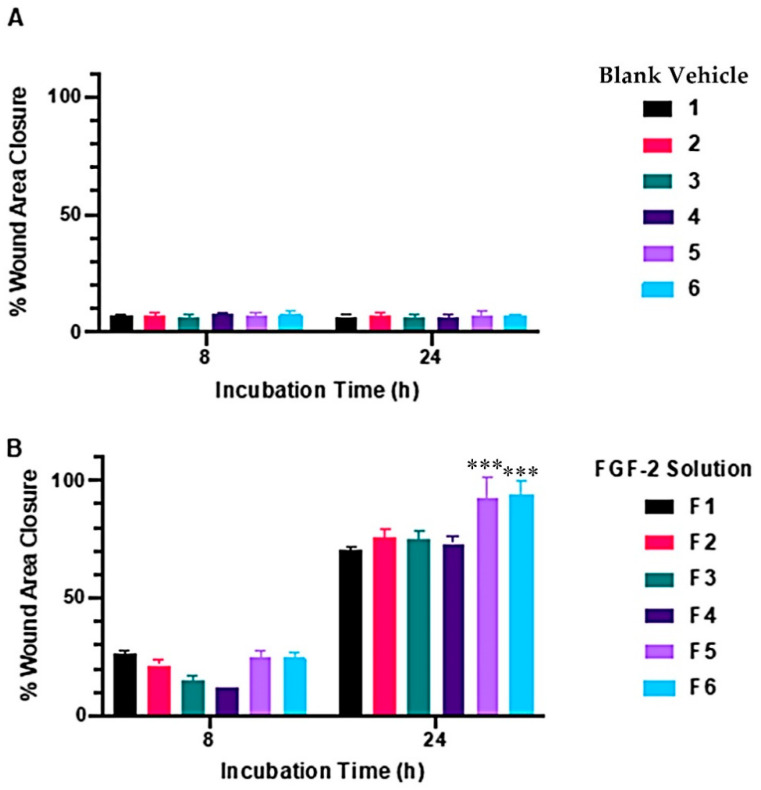
Wound-healing capacity of blank vehicles (**A**) and FGF-2 solutions (**B**) measured by percent wound area recovery of a confluent fibroblast monolayer. Blank vehicle: 1 (water only), 2 (methylcellulose (MC) 0.05% *w*/*v*), 3 (alanine 20 mM), 4 (human serum albumin (HSA) 1 mg/mL), 5 (MC 0.05% *w*/*v* and alanine 20 mM) and 6 (MC 0.05% *w*/*v* and HSA 1 mg/mL). FGF-2 solutions (F1–F6) contained 50 ng/mL FGF-2 in the corresponding vehicles. Data represent mean ± SD (*n* = 3). The triple asterisks (***) denote a highly significant increase in cell migration for F5 and F6, with a *p*-value of ≤0.001 when compared to their corresponding blank vehicles.

**Figure 3:**
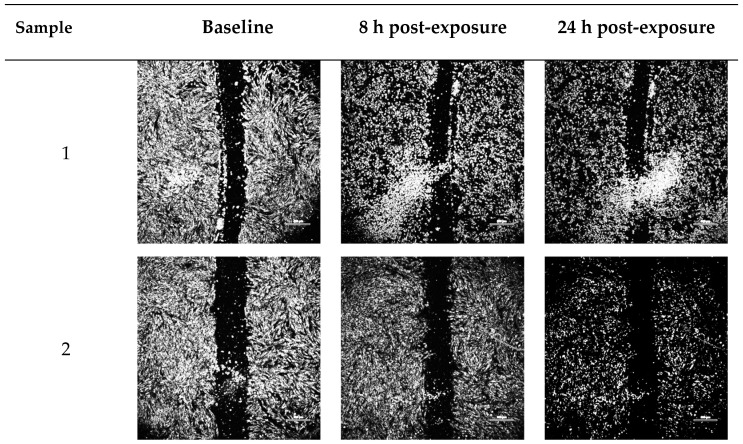

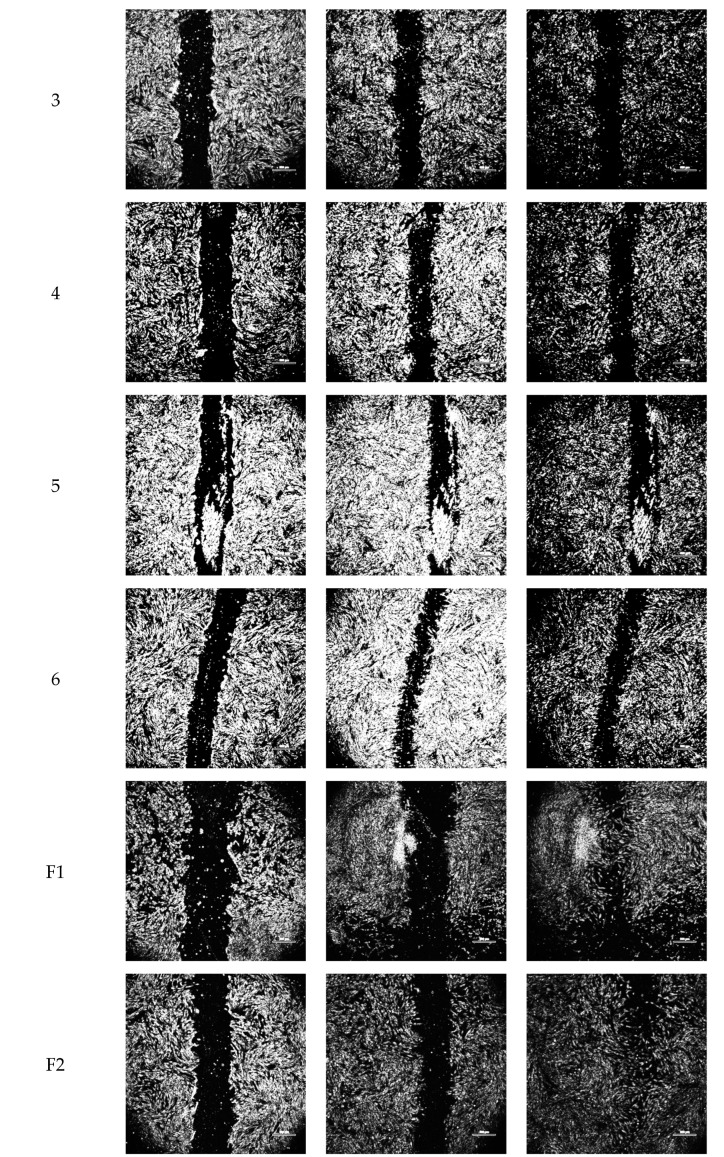

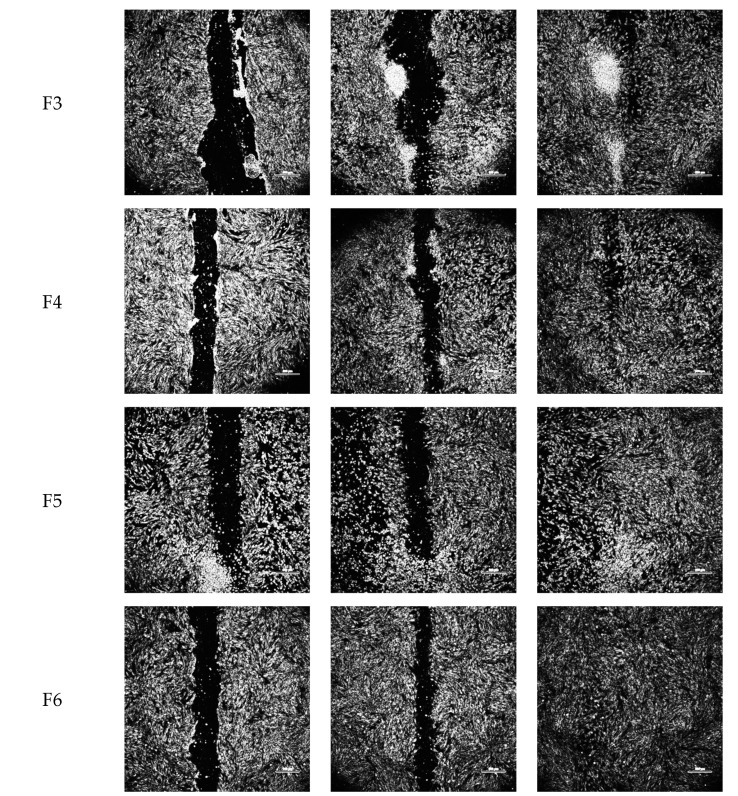
Representative optical micrographs of wound area recovery of human dermal fibroblast monolayers at baseline, and after 8 h and 24 h post-exposure to blank vehicles and FGF-2 solutions. Blank vehicle: **1** (water only), **2** (methylcellulose (MC) 0.05% *w*/*v*), **3** (alanine 20 mM), **4** (human serum albumin (HSA) 1 mg/mL), **5** (MC 0.05% *w*/*v* and alanine 20 mM) and **6** (MC 0.05% *w*/*v* and HSA 1 mg/mL). FGF-2 solutions (F1–F6) contained 50 ng/mL FGF-2 in the corresponding vehicles. Magnification: 100×, scale bar = 500 µm. F1 (control), F2 (with methylcellulose (MC) 0.05% *w*/*v*), F3 (with alanine 20 mM), F4 (with human serum albumin (HSA) 1 mg/mL), F5 (with MC 0.05% *w*/*v* and alanine 20 mM) and F6 (with MC 0.05% *w*/*v* and HSA 1 mg/mL).

In vitro cellular regenerative efficacy was measured by exposing the simulated wound of the dermal fibroblast monolayer to the blank vehicles and FGF-2 solutions (50 ng/mL). Figure 2 shows the percent wound area closure while Figure 3 shows representative microscope images of the cell monolayers at baseline, and at 8 h and 24 h post-exposure. The six blank vehicles achieved very low wound area closure rates, only 5–9% after 24 h, indicating their minimal capacity to stimulate cellular regeneration in the absence of FGF-2. The FGF-2 solutions showed various degrees of wound closure enabling that was dependent on the excipient present in the solutions. At 8 h post exposure, F1, F2, F5 and F6 displayed comparable wound closure rates that were greater (*p* < 0.05) than those of F3 and F4. However, at 24 h post exposure, F2, F3 and F4 were no more effective than the control F1, achieving closure rates of 75.7, 74.9, 73.2%, respectively, compared to 70.5% for F1. By comparison, F5 and F6 continued to show significantly enhanced activity (*p* < 0.0001), producing wound closure rates of 92.5% and 94.1%, respectively, at 24 h.

### 2.3. Chemotactic Migration Assay

**Figure 4:**
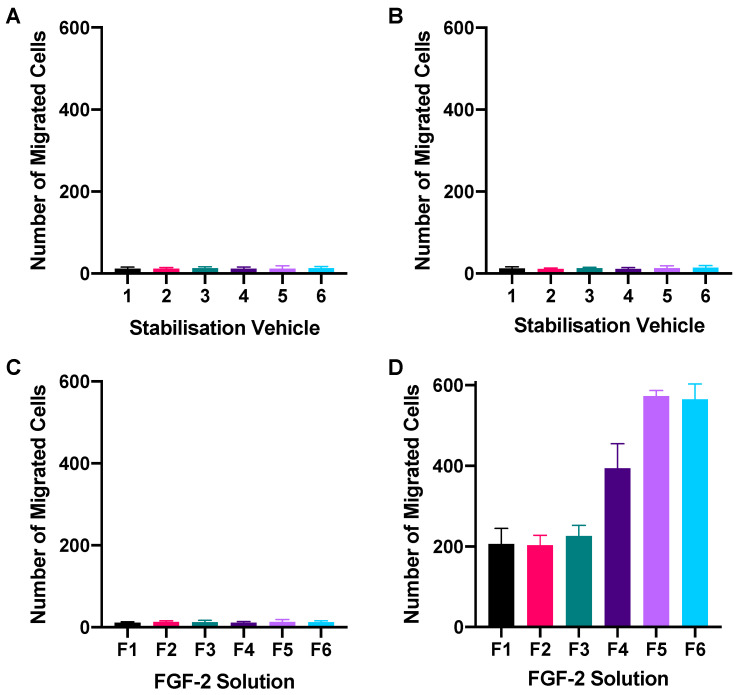
Chemotactic migration of human dermal fibroblasts following 24 h exposure to blank vehicles (1–6) applied in both the apical and basolateral chambers (**A**), or in basolateral chamber only (**B**); and FGF-2 solutions (F1–F6) applied in both the apical and basolateral chambers (**C**), or in basolateral chamber only (**D**) of a transwell set-up. Blank vehicles: 1 (water only), 2 (methylcellulose (MC) 0.05% *w*/*v*), 3 (alanine 20 mM), 4 (human serum albumin (HSA) 1 mg/mL), 5 (MC 0.05% *w*/*v* and alanine 20 mM) and 6 (MC 0.05% *w*/*v* and HSA 1 mg/mL); and FGF-2 solutions (F1–F6) containing 50 ng/mL FGF-2 in the corresponding vehicles. Data represent mean number of migrated cells/well ± SD (*n* = 3).

**Figure 5:**
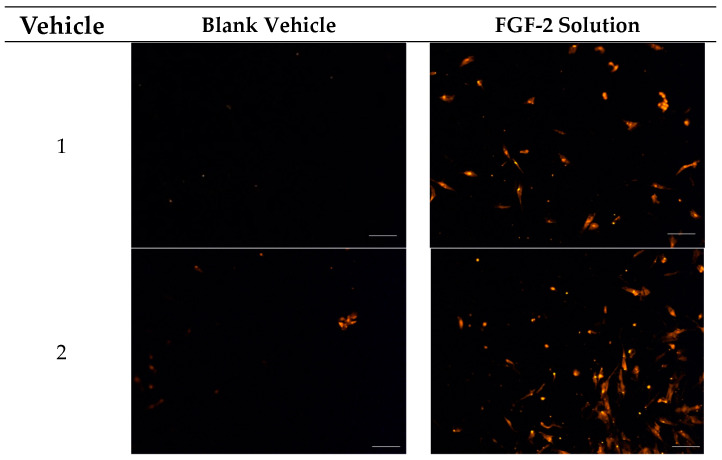

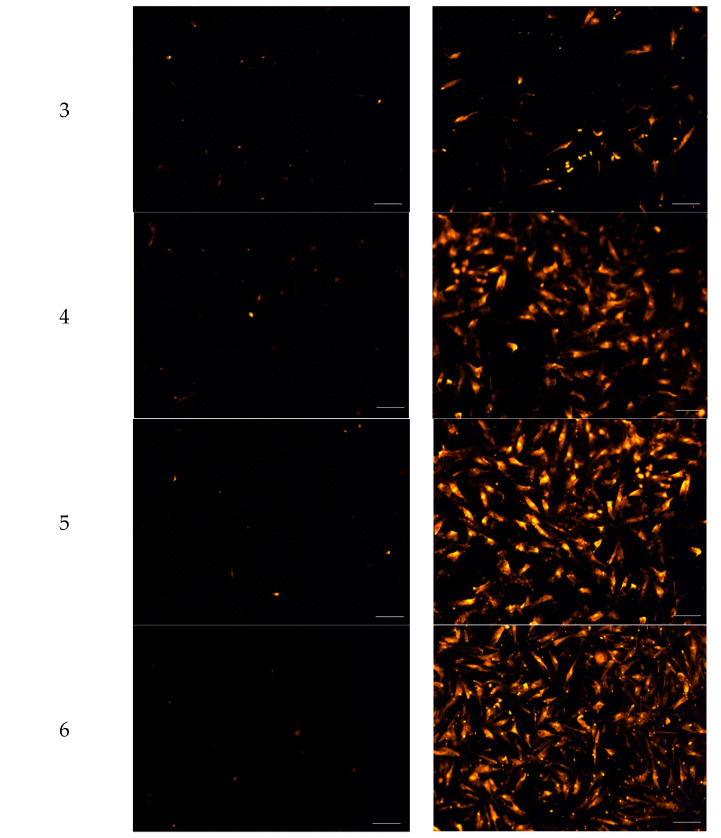
Fluorescence micrographs showing chemotactic migration of human dermal fibroblast cells to the basal surface of a transwell membrane in response to FGF-2. Cells seeded on the apical transwell membrane were exposed to FGF-2 solutions or blank vehicles added to the basolateral chamber. Samples comprise blank vehicles or FGF-2 (50 ng/mL in the vehicle) in the vehicles: 1 (water only), 2 (methylcellulose (MC) 0.05% *w*/*v*), 3 (alanine 20 mM), 4 (human serum albumin (HSA) 1 mg/mL), 5 (MC 0.05% *w*/*v* and alanine 20 mM) and 6 (MC 0.05% *w*/*v* and HSA 1 mg/mL). Magnification: 200×, scale bar = 100 µm.

A transwell setup was used to determine whether FGF-2 in the presence of the stabilizers retained its chemoattractant characteristics. Figure 4 shows the number of fibroblasts that had migrated to the basal transwell membrane in response to FGF-2 application while Figure 5 shows representative fluorescence micrographs of the membrane. None of the blank vehicles, whether applied to only the basolateral chamber or to both the apical and basolateral chambers of the transwells, caused more than 20 cells to migrate to the basal membrane (Figure 4A,B). FGF-2 samples (F1–F6, 50 ng/mL) added to both the apical and basolateral chambers also did not result in significant number of migrated cells (*p* = 0.8265) (Figure 4C). In contrast, FGF-2 samples added to only the basolateral chamber led to a cellular migration count that was 10- to 30-fold higher than the numbers obtained with the corresponding vehicles (*p* < 0.0001) (Figure 4D). This indicated that FGF-2 in F1–F6 exhibited chemoattractant behavior. A comparison of the FGF-2 samples indicated that F5 and F6 produced the strongest chemoattractant effects, and both formulations were again comparable in their effectiveness.

## 3. Discussion

The cytoproliferative, cellular migratory and chemoattractive effects of FGF-2 make it an attractive potential therapeutic agent for wound products and tissue engineering constructs. However, the rapid degradation of FGF-2 in solution has to be addressed in order to develop acceptable patient-centric FGF-2 medicinal products. We have achieved FGF-2 stabilization in solution by incorporating the excipients, MC (0.05%), alanine (20 mM) and HSA (1 mg/mL) and have found the combination of MC with alanine (F5) and MC with HSA (F6) to be particularly effective. F5 and F6 were also amenable to freeze drying, and the resultant lyophilized powders were stable to storage at −4 °C, 4 °C and 18 °C for up to 12 months [28]. Moreover, lyophilized powders are readily reconstituted into solutions by the addition of water, and the reconstituted FGF-2 solutions were stable for at least 7 days at 4 °C and 18 °C, which represents a significant breakthrough as the FGF-2 solutions can then be readily stored in hospital wards and homes for administration to patients. The chemical stability of FGF-2 in F5 and F6 was established via ELISA [28]; however, it is not yet known whether the addition of MC, alanine, HSA, alone or in combination, would affect the beneficial biological effects of FGF-2. The in vitro cell-based data generated in this study showed that F5 and F6 not only retained the cellular activity of FGF-2 but were superior to the other FGF-2 solutions applied in this study.

FGF-2′s role as a growth factor was validated in this study as all the FGF-2 solutions consistently outperformed the corresponding blank vehicles in cellular responses. In the absence of a stabilizing excipient, however, the cellular effects of FGF-2 might not be fully realised, as shown by the control F1. Of all the FGF-2 formulations, F1 exhibited the weakest responses, producing a detectable cellular proliferative effect only at high FGF-2 doses, and also having the highest EC_50_ and the lowest maximal effect on the human dermal fibroblasts. These findings, together with the observation that F1, while showing an early promise in closing the wound area of the fibroblast monolayer could not attain complete wound closure, support our previous work that FGF-2 is rapidly inactivated in solution in the absence of stabilizers.

FGF-2 in solution in the absence of a stabilizer is reported to have a half-life of only 37 min at 37 °C [28,29], and for F1, only 30 min [28]. The endogenous FGF-2 stabilizer is heparin, (27) but heparin is pharmacologically active and could not be safely applied to stabilize FGF-2 medicinal products. We chose to incorporate GRAS excipients commonly used in medicinal products as stabilizers, and found MC, alanine, and HSA to have different effectiveness at stabilizing FGF-2 at 37 °C. When applied alone, MC, alanine and HSA could not completely mitigate the degradation of FGF-2 at 37 °C; by contrast, a combination of MC and alanine (F5) or MC and HSA (F6) provided complete protection for the bioactive FGF-2 content over 2 h at 37 °C, while F5 also retained 97% residual FGF-2 after 8 h at 37 °C (F6 showed 50% residual FGF-2 under the same conditions) [28]. The cytoproliferative dose-responses on the human dermal fibroblasts were in the ranking order of F5~F6 > F2 > F4~F3 > F1, which was in general agreement with the residual bioactive FGF-2 content measured in these FGF solutions after 2 h at 37 °C [28]. Between F5 and F6, the greater stability of F5 at 37 °C might explain its greater cytoproliferative effects at lower FGF-2 concentrations. However, both F5 and F6 elicited comparable cellular responses at higher FGF-2 doses, and at 50 ng/mL, were able to cause >90% closure of the wound area in the human dermal fibroblast monolayer after 24 h.

The maximal cytoproliferative activity of F2, F3, F4, F5 and F6 were higher than F1, which might suggest that the effects of FGF-2 on the human dermal fibroblasts was potentiated by the stabilizers. MC is reported to stimulate the proliferation of human skin cells (NCTC 135) and monkey kidney cells (LLCMK2), but not murine fibroblast cells (2571+L) [30,31]. There is evidence that alanine and HSA support lymphocyte proliferation [32] and mammalian cell growth [33], respectively, but the effects of alanine and HSA on fibroblast proliferation have not been reported. Data obtained for the blank vehicles in this study indicate that MC, alanine, and HSA, in the absence of FGF-2, do not support the proliferation and migration of the human dermal fibroblasts. Thus, the enhanced cellular responses of F2, F3, F4, and particularly F5 and F6, could be attributed to the stabilization of FGF-2 in these solutions.

FGF-2 in the stabilized solutions acted as a chemoattractant to the human dermal fibroblast cells, similar to its effects on other cell types [34,35]. F4, which contained only HSA as stabilizer, showed stronger chemoattractive effects than F1, F2 and F3, suggesting that HSA could promote cell migration. HSA was also present in F6, but not F5, yet both F5 and F6 showed comparable chemotactic effects that were stronger than F4, suggesting that ultimately, the cellular effects of the FGF-2 solutions were determined by the stability of the growth factor. The integrity of FGF-2 in fully stabilized solutions enables higher bioactivity per applied dose, and also sustained cytoproliferative, cellular migratory and chemoattractive effects over a longer period of time.

Chronic wounds often have reduced concentrations of growth factors, including FGF-2, that then adversely affect cellular proliferation, migration, differentiation, and angiogenesis, and prevent the healing and re-vascularization of wounds [36,37]. Data from this study demonstrate that the effective stabilization of FGF-2 can potentiate cellular responses, with FGF-2 stabilized by MC in combination with alanine (F5) or MC in combination with HSA (F6) providing the greatest benefits for human dermal fibroblasts.

Our study applied human dermal fibroblasts to validate the cytoproliferative, migratory, and chemoattractive effects of stabilized FGF-2 formulations. This cell-culture choice was strategic, aiming to establish a base understanding of FGF-2′s therapeutic actions in a controlled, reproducible setting, given the pivotal role of human dermal fibroblasts in wound healing and their proven response to growth factors. The tympanic membrane has structural similarity to the dermal layer, particularly its fibrous middle layer that is rich in fibroblasts, and this further underpins the rationale for this approach. By demonstrating that stabilized FGF-2 enhances the proliferation and migration of human dermal fibroblasts, we indirectly substantiate its potential for tympanic membrane repair, and address the chronic TMP challenges like recurrent infections and hearing loss. Our findings suggest stabilized FGF-2 has promising therapeutic application for TMP, backed by its established bioactivity and efficacy in promoting key regenerative processes, which merits further tympanic membrane-specific research to explore direct benefits. This study is intended as a pilot investigation, with a comprehensive characterization of fibroblasts in primary cultures envisioned for future studies to ensure the development of targeted, effective treatment modalities.

## 4. Materials and Methods

### 4.1. Materials

Phosphate buffered saline (PBS), penicillin/streptomycin 100× (P/S), formaldehyde 4% and propidium iodide (PI) were from Solarbio Science and Technology Co. (Beijing, China), fetal bovine serum (FBS) was from Lonsera (Shanghai, China), Dulbecco’s Modified Eagle Medium (DMEM) was from Gibco (Grand Island, NY, USA)) and cell counting kit-8 (CCK-8) was from Dojindo Molecular Technologies (Shanghai, China). Human recombinant FGF-2 was from Peprotech (Suzhou, China) and detected using the human FGF-2 ELISA kit from Thermo Fisher Scientific (Waltham, MA, USA). Methylcellulose USP 4000 was from the Professional Compounding Chemists of America (PCCA; NSW, Australia), while human serum albumin, DL-alanine and hydroxypropyl methylcellulose (HPMC) were from Sigma-Aldrich (St Louis, MO, USA). Deionized water was used throughout.

### 4.2. Sample Preparation and Quantification by ELISA

Bioactive FGF-2 content in the commercial lyophilized FGF-2 powder was quantified by ELISA, and the information used to determine the powder mass for preparing formulations to contain 1600 ng/mL bioactive FGF-2. Formulations F1–F6 were prepared using the specified vehicles according to Table 1. Corresponding vehicles without FGF-2 (1–6) served as blank controls. The vehicles comprised water alone or water with 0.05% *w*/*v* methylcellulose (MC), 20 mM alanine or 1 mg/mL human serum albumin (HSA), added alone or in combination to stabilize FGF-2 [23]. Samples for cell-culture experiments were prepared by serially diluting the FGF solutions or blank vehicles with test culture medium (TCM, comprising DMEM with 1% FBS and 1% P/S) to give the working concentrations required.

### 4.3. Cell Culture

Primary human dermal fibroblasts were isolated from skin donated, with informed consent, by a healthy adult volunteer in accordance with the protocols of the Affiliated Hospital of Guilin Medical University. The fibroblasts were seeded at a density of approximately 2.2 × 10^6^ cells in 10 mL of complete culture media (CCM, comprising DMEM with 10% FBS and 1% P/S) in a 100 mm culture dish (Eppendorf, Hamburg, Germany) and cultured till confluence at 37 °C under 5% CO_2_ (Forma Series II Water Jacket CO_2_ Incubator, Thermo Scientific, Waltham, MA, USA) before they were used for further experiments.

The choice of primary human dermal fibroblasts for this study was grounded in their well-documented responsiveness to fibroblast growth factors and their critical role in wound-healing processes. There is currently no in vitro TMP model. Human dermal fibroblasts offer a reproducible model to assess the cytoproliferative and chemotactic effects of FGF-2, providing a foundational understanding crucial for the future development and application of FGF-2 formulations in tissue repair and regeneration. By recognizing the importance of cellular characterization in primary cultures, this approach allows us to establish a baseline for FGF-2′s therapeutic potential, acknowledging that detailed fibroblast characterization is essential for tailoring future drug applications.

### 4.4. Dose-Response Assay

Fibroblasts (5 × 10^3^ cells in 100 µL of CCM per well) were cultured for 24 h in 96-well plates (Corning, New York, NY, USA). Cell growth was arrested by replacing the medium with TCM and incubating the cells for 24 h. FGF-2 samples from F1 to F6 were applied in escalating doses (0.0098–200 ng/mL) and this was mirrored by application of the corresponding blank vehicles as controls. After 48 h, the FGF-2 and blank samples were replaced with 100 µL of CCK-8 which had been diluted 1:10 (*v*/*v*) with TCM, and the absorbance in each well was measured after 1 h at 450 nm (FilterMax F5 Multi-Mode plate reader, Molecular Devices, San Jose, CA, USA). The difference in absorbance between wells containing the FGF-2 solution and the respective vehicle was plotted against the FGF-2 dose, and the half maximal effective concentration (EC50) for each FGF-2 solution was determined using a 4-parameter logistic fit (GraphPad Prism 8, La Jolla, CA, USA).

### 4.5. In Vitro Wound-Healing Assay

FGF-2′s capacity for cellular regeneration was investigated via an in vitro simulated wound healing assay. Fibroblasts (2 × 10^4^ cells/well) cultured with 1 mL CCM for 48 h to confluency in 24-well plates (Corning, New York, NY, USA) were cultured for another 24 h with 1 mL TCM to arrest cell growth. A ‘wound’ was created in the confluent cell monolayer using a sterilized pipette tip (200 µL) and the dislodged cells removed by washing twice with PBS. FGF-2 solutions (F1–F6, 1 mL, 50 ng/mL FGF-2) and blank vehicles (1–6, 1 mL) were then applied to the wound area. Microscopic images (Nikon Eclipse Ti-S attached with Nikon DS-Ri2 camera and NIS Elements v5.01 software, Nikon Corporation, Tokyo, Japan) were taken at 0, 8 and 24 h, and processed by ImageJ to determine the wound area and the rate of wound closure, calculated as a percent of the baseline wound area measured at 0 h. Experiments were performed in triplicate.

### 4.6. Chemotactic Migration Assay

Chemotactic migration of the fibroblasts in response to FGF-2 was assessed using the Boyden chamber technique [37]. Fibroblasts (5 × 10^4^ cells in 200µL of TCM) were seeded onto the apical membrane of 24-transwell plates (8 µm pore size; Corning, New York, NY, USA) with 500 µL of TCM in the basolateral chamber. Following cell attachment at 24 h incubation, FGF-2 samples (F1-F6; 50 ng/mL) and corresponding vehicle samples (1–6) were applied to replace the TCM in the basolateral chamber only (500 µL), or in both the apical and basolateral chambers (200 µL and 500 µL, respectively). FGF-2 samples added to only the basolateral chamber would create a concentration gradient across the membrane allowing for chemotactic migration of cells to be detected. In contrast, the addition of samples to both the basolateral and apical chambers would equalize concentrations across the membrane and control for any chemokinetic migration of the cells. After 24 h incubation, residual cells on the apical membrane (non-migratory) were carefully removed with a cotton swab soaked in PBS, and non-adherent cells were removed by flushing the wells with PBS. Cells that had migrated and attached to the basal surface of the membrane were fixed in 4% formaldehyde, stained with 10 µM propidium iodide and visualized by fluorescence microscopy (excitation 535 nm, emission 615 nm; Leica DM4 B; Leica Microsystems, Wetzlar, Germany). To ensure as many cells as possible were included in the count, 7 images were obtained for each membrane and analyzed using ImageJ to determine the total number of cells which had migrated. Experiments were performed in triplicate and the cell count expressed as mean ± SD.

### 4.7. Data Analysis

Results are expressed as mean ± SD. Data were analyzed by two-way ANOVA with post hoc Tukey’s test (GraphPad Prism 8, La Jolla, CA, USA) applied for paired comparison of means, unless stated otherwise. A *p*-value ≤ 0.05 was considered to be significant.

## 5. Conclusions

This investigation revealed that FGF-2 formulations F5 and F6 exhibited exceptional bioactivity and therapeutic potential for chronic tympanic membrane perforation (TMP) treatment, suitable for use as both aqueous solutions and lyophilized powders. Their stability and enhanced bioactivity make them ideal for advancing into scaffold-based therapies, addressing the tympanic membrane’s unique challenges that hinder cellular repair and regeneration. Consequently, the formulations of F5 and F6 exhibit promising properties for wound healing applications, with a potential for future exploration in TMP treatment based on their demonstrated stability and bioactivity. Further in vivo studies and TMP-specific research are necessary to fully ascertain their therapeutic potential and applicability for TMP repair.

## 6. Patents

This work is the subject of Patent Application AU 2021902450.

## Figures and Tables

**Table 1:** Blank vehicles and FGF-2 formulations prepared using the corresponding vehicle. FGF formulations contained 1600 ng/mL of FGF-2 as determined by ELISA.

Vehicle Constituents	Notation for Blank Vehicle	Notation for Solutions Containing 1600 ng/mL Bioactive FGF-2 in Vehicle
Water (control)	1	F1
Methylcellulose (MC; 0.05% *w*/*v*) in water	2	F2
Alanine (20 mM) in water	3	F3
Human serum albumin (HSA; 1 mg/mL) in water	4	F4
MC (0.05% *w/v*) and alanine (20 mM) in water	5	F5
MC (0.05% *w*/*v*) and HSA (1 mg/mL) in water	6	F6

## Data Availability

Data is contained within the article.
