# Correction: Benington et al. In Vitro Assessment of Wound-Healing Efficacy of Stabilized Basic Fibroblast Growth Factor (FGF-2) Solutions. *Pharmaceuticals* 2024, *17*, 247

**DOI:** 10.3390/ph17070892

**Published:** 2024-07-05

**Authors:** Leah Benington, Jingxin Mo, Mingxin Li, Gunesh Rajan, Cornelia Locher, Lee Yong Lim

**Affiliations:** 1 Division of Pharmacy, School of Allied Health, University of Western Australia, Perth, WA 6009, Australia; leah.benington@uwa.edu.au (L.B.); connie.locher@uwa.edu.au (C.L.); 2 Neuroscience Laboratory, The Affiliated Hospital of Guilin Medical University, Guilin 541001, China; jingxin.mo@hotmail.com (J.M.); mingxinli822@163.com (M.L.); 3 Graduate School of Biomedical Engineering, University of New South Wales, Sydney, NSW 2052, Australia; 4 Department of Pharmacy, Tangshan Central Hospital, Tangshan 063000, China; 5 Otolaryngology, Head & Neck Surgery, Division of Surgery, Medical School, University of Western Australia, Perth, WA 6009, Australia; gunesh.rajan@luks.ch; 6 Department of Otolaryngology, Head & Neck Surgery, Luzerner Kantonsspital, 6000 Luzern, Switzerland

In the original publication [1], there was a mistake in Figure 5. which was published. The image for the FGF-2 solution in vehicle 5 was mislabeled and required a replacement. A corrected version of Figure 5 appears below. The authors confirm that the scientific conclusions are unaffected. This correction was approved by the Academic Editor. The original publication has also been updated.

## Figures and Tables

**Figure 5:**
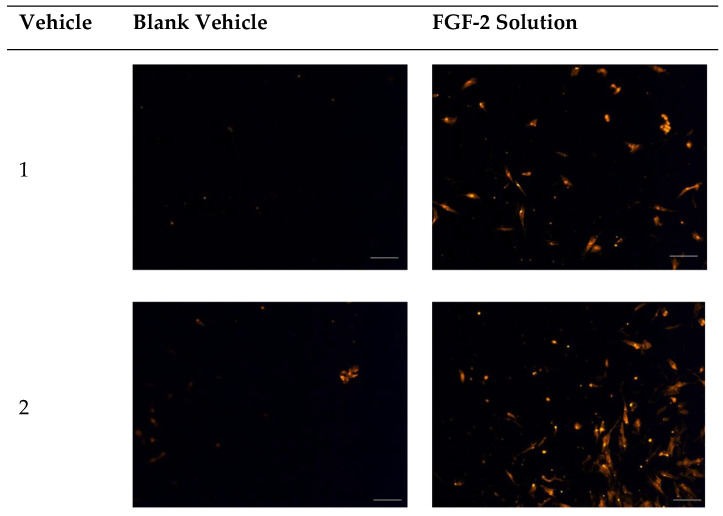

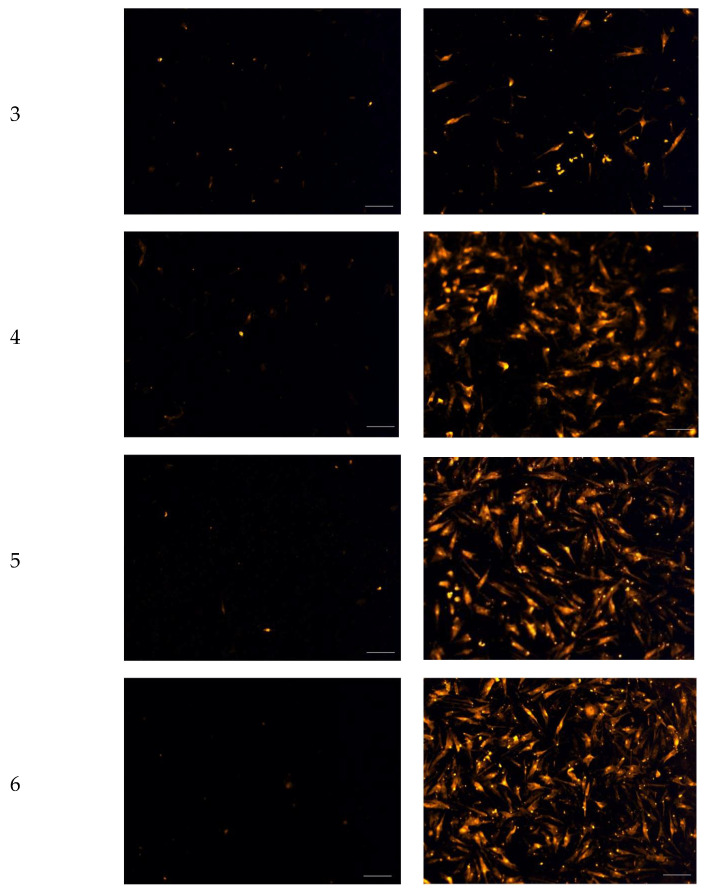
Fluorescence micrographs showing chemotactic migration of human dermal fibroblast cells to the basal surface of a transwell membrane in response to FGF-2. Cells seeded on the apical transwell membrane were exposed to FGF-2 solutions or blank vehicles added to the basolateral chamber. Samples comprise blank vehicles or FGF-2 (50 ng/mL in the vehicle) in the vehicles: 1 (water only), 2 (methylcellulose (MC) 0.05% *w*/*v*), 3 (alanine 20 mM), 4 (human serum albumin (HSA) 1 mg/mL), 5 (MC 0.05% *w*/*v* and alanine 20 mM) and 6 (MC 0.05% *w*/*v* and HSA 1 mg/mL). Magnification: 200×, scale bar = 100 µm.

## References

[1] Benington L., Mo J., Li M., Rajan G., Locher C., Lim L.Y. (2024). In Vitro Assessment of Wound-Healing Efficacy of Stabilized Basic Fibroblast Growth Factor (FGF-2) Solutions. Pharmaceuticals.

